# Academic Emergency Medicine Faculty Experiences with Racial and Sexual Orientation Discrimination

**DOI:** 10.5811/westjem.2020.6.47123

**Published:** 2020-08-21

**Authors:** Dave W. Lu, Ava Pierce, Joshua Jauregui, Sheryl Heron, Michelle D. Lall, Jennifer Mitzman, Danielle M. McCarthy, Nicholas D. Hartman, Tania D. Strout

**Affiliations:** * University of Washington School of Medicine, Department of Emergency Medicine, Seattle, Washington; † University of Texas Southwestern Medical School, Department of Emergency Medicine, Dallas, Texas; ‡ Emory University School of Medicine, Department of Emergency Medicine, Atlanta, Georgia; § The Ohio State University College of Medicine, Department of Emergency Medicine, Columbus, Ohio; ¶ Northwestern University Feinberg School of Medicine, Department of Emergency Medicine, Chicago, Illinois; || Wake Forest School of Medicine, Department of Emergency Medicine, Winston-Salem, North Carolina; # Tufts University School of Medicine – Maine Medical Center, Department of Emergency Medicine, Portland, Maine

## Abstract

**Introduction:**

Despite the increasing diversity of individuals entering medicine, physicians from racial and sexual minority groups continue to experience bias and discrimination in the workplace. The objective of this study was to determine the current experiences and perceptions of discrimination on the basis of race and sexual orientation among academic emergency medicine (EM) faculty.

**Methods:**

We conducted a cross-sectional survey of a convenience sample of EM faculty across six programs. Survey items included the Overt Gender Discrimination at Work (OGDW) Scale adapted for race and sexual orientation, and the frequency and source of experienced and observed discrimination. Group comparisons were made using t-tests or chi-square analyses, and relationships between race or sexual orientation, and we evaluated physicians’ experiences using correlation analyses.

**Results:**

A total of 141 out of 352 (40.1%) subjects completed at least a portion of the survey. Non-White physicians reported higher mean racial OGDW scores than their White counterparts (13.4 vs 8.6; 95% confidence interval (CI) for difference, −7.7 – −2.9). Non-White EM faculty were also more likely to report having experienced discriminatory treatment based on race than were White EM faculty (48.0% vs 12.6%; CI for difference, 16.6% – 54.2%), although both groups were equally likely to report having observed race-based discrimination of another physician. EM faculty who identified as sexual minorities reported higher mean sexual minority OGDW scores than their heterosexual counterparts (11.1 vs 7.1; 95% CI for difference, −7.3 – −0.6). There were no significant differences between sexual minority and heterosexual faculty in their reports of experiencing or observing discrimination based on sexual orientation.

**Conclusion:**

EM faculty from racial and sexual minority groups perceived more discrimination based on race or sexual orientation in their workplace than their majority counterparts. EM faculty regardless of race or sexual orientation were similar in their observations of discriminatory treatment of another physician based on race or sexual orientation.

## INTRODUCTION

Approximately half of all students enrolled in United States medical schools in 2019 self-reported as non-White.^1^ Despite the increasing diversity of individuals entering medicine, physicians from racial minority groups continue to experience racial bias and discrimination in the workplace, including disparities in career satisfaction, job turnover, federal research grants, and academic promotion.^2^–^7^ Many studies have detailed racial discrimination of minority medical students and physicians.^2^,^6^,^8^,^9^ There is currently little data describing racial discrimination in academic emergency medicine (EM).^10^–^12^ A better understanding of the current workplace environment with regard to racial discrimination will aid efforts to promote equity, inclusion, and diversity within the emergency physician workforce.

Many physicians who identify as lesbian, gay, bisexual, or transgender (LGBT) also report workplace harassment, social ostracization, and discriminatory treatment.^13^,^14^ A significant proportion of LGBT physicians, trainees, and medical students cited concerns of discrimination and harassment for their need to conceal their sexual or gender identities.^15^–^18^ LGBT providers’ discomfort with this disclosure is one contributor to their higher levels of distress, burnout, and depression compared to their heterosexual colleagues.^14^–^16^,^19^ Few studies have examined the experiences with workplace discrimination among physicians who identify as sexual minorities.^13^,^14^,^20^ Current data on this understudied provider population will fill an important knowledge gap and inform the aforementioned diversity efforts of both EM and healthcare in general.

The objective of this study was to determine the current experiences and perceptions of discrimination by race and sexual orientation among academic EM faculty. We hypothesized that racial and sexual minority emergency physicians would have greater perceptions of and more experiences with discrimination compared to their non-minority colleagues.

## METHODS

### Study Design

This study was a cross-sectional survey of a convenience sample of EM faculty on their perceptions of and experiences with racial and sexual identity discrimination in the workplace. Data from the same study examining the experiences of EM faculty with workplace gender discrimination have been presented previously.^21^ Details of the same methodology are summarized and briefly presented here.

### Study Setting and Population

All EM faculty, except the study authors, at six urban, academic training programs were eligible for this study. Study sites were departments of EM located in the following regions: New England (one); Southeast (two); South (one); Midwest (one); West (one). The survey was administered over February–March 2019.

**Population Health Research Capsule**

What do we already know about this issue?

Studies have shown that physicians from racial and sexual minority groups experience bias and discrimination in the workplace.

What was the research question?

What are the experiences of academic EM faculty with racial and sexual orientation discrimination in the workplace?

What was the major finding of the study?

Racial and sexual minority faculty perceived greater discrimination based on race and sexual orientation than their peers.

How does this improve population health?

There is cultural momentum to confront discrimination based on race and sexual orientation. Efforts to promote equity and diversity within the emergency physician workforce are needed.

### Study Protocol

An anonymous electronic survey was emailed to all eligible subjects. Subjects consented to the voluntary study by completing the survey on an online, secure platform. Three reminder emails were sent to non-responders. The study was either approved or deemed exempt from review by each site’s institutional review board.

### Measurements

No single, well-validated instrument could be found that satisfactorily measured the multiple aspects of workplace racial and sexual identity discrimination that were of interest. Based on a review of the current literature, we created a 31-item survey composed of questions adapted from surveys used in similar work among populations of physicians from multiple specialties. The survey was pre-tested by EM faculty at five institutions to ensure respondent comprehension.

We measured subjects’ perceptions of discrimination using five questions adapted from the Overt Gender Discrimination at Work (OGDW) Scale, an instrument that assesses the perception of gender biases in the workplace, by substituting references to gender with race or sexual identity.^22^,^23^ The scale asks: “How strongly do you agree with the following statements about your current place of work?: 1) I have been treated unfairly at work because of my [race or sexual orientation]; 2) The people I work with sometimes make [racist or anti-LGBTQ] statements and/or decisions; 3) I feel that some of the policies and practices of this organization are [racist or anti-LGBTQ]; 4) At work, I sometimes feel that my [race or sexual orientation] is a limitation; and 5) At work, I do not get enough recognition because of my [race or sexual orientation]. Responses are based on a 1–5 Likert scale, with 1 = strongly disagree; 3 = neutral; and 5 = strongly agree. Scores range from 5–25, with higher scores indicating higher perceptions of discrimination.

Using questions adapted from prior work,^24^ we also asked subjects to report the frequency with which they have *experienced* discriminatory treatment based on their race or sexual orientation, as well as the frequency with which they have *observed* such discriminatory treatment of another physician. Responses included weekly, monthly, annually, rarely, and never. Those respondents who reported weekly, monthly, or annually to either experiencing discriminatory treatment or having observed discriminatory treatment of another physician based on race or sexual orientation were subsequently asked to identify the source of the discriminatory treatment. Sources included the following: university / medical school / hospital administrator; consulting or admitting physician; EM attending physician; resident physician; medical student; nursing staff; clerical staff; emergency medical services personnel; patient; and other. Subjects were asked to report the frequency with which they had experienced or had observed discriminatory treatment from each source (weekly, monthly, annually, rarely, and never). Developed by Bruce and colleagues,^24^ these items were designed to categorize the scope, type, and source of gender-based discrimination in medicine. We substituted gender with race or sexual identity for purposes of this study.

We collected limited demographic information (Table 1) to prevent easy identification of otherwise anonymous responses and to encourage honest reporting. We did not obtain information linking subjects by study site.

### Data Analysis

Data were collected electronically using Qualtrics (Qualtrics, Provo, UT) software and exported into SPSS for Windows v25.0 (SPSS, Inc., Chicago, IL) for analysis. Continuous variables (eg, age, modified OGDW scores) were examined for normality using visual inspection of histograms, P-P plots, and Pearson’s skewness statistic. We used the t-test for independent samples to compare group means for continuous variables. In addition, Pearson’s chi-square analysis or Fisher’s exact test was employed to compare proportions across categorical variables. In some cases, for example, in categorizing respondents as having experienced or observed racial or sexual orientation discrimination, response categories were collapsed into dichotomous categories a priori to aid in result interpretation (“never” and “rarely” vs “weekly,” “monthly,” and “annually”). Similarly, the anticipated small numbers of racial and sexual minority participants (Table 1) necessitated a priori collapse of these individual response categories into dichotomous variables (eg, non-White vs White, sexual minority vs non-minority) to aid analysis. To assess the strength and direction of relationships between variables, we used Pearson’s correlation coefficient or Spearman’s rho, as appropriate for the data. Partial correlations were also used to evaluate relationships between variables, while controlling for the effect of a covariate (race or gender orientation). Data are presented as frequencies, proportions, means and 95% confidence intervals (CI) around differences between means. All p-values are two-tailed and we accepted an alpha of less than 0.05 as statistically significant.

## RESULTS

A total of 141 out of 352 (40.1%) subjects completed at least a portion of the survey. Respondents were mostly male (n = 80, 61.1%) and White (n = 104, 79.4%) (Table 1). The mean age reported by participants was 41.3 years (range 30–64 years) with the majority of respondents (n = 73, 55.7%) having completed residency training within 10 years. In contrast, according to 2017 data from the Association of American Medical Colleges (AAMC), 72.4% of active emergency physicians in the US were male,^25^ with 65.2% under 55 years of age.^25^ In addition, 2018 AAMC data of full-time US medical school faculty revealed that 63.9% were White, 3.6% were Black or African American, 3.2% were Hispanic or Latino, 19.3% were Asian or Pacific Islander, and 0.2% were American Indian or Alaska Native.^26^ Although our sample appears to be younger, less male, and more White than national samples, direct comparisons were not possible due to differences in queried age and racial / ethnic categories.

**Figure 1:**
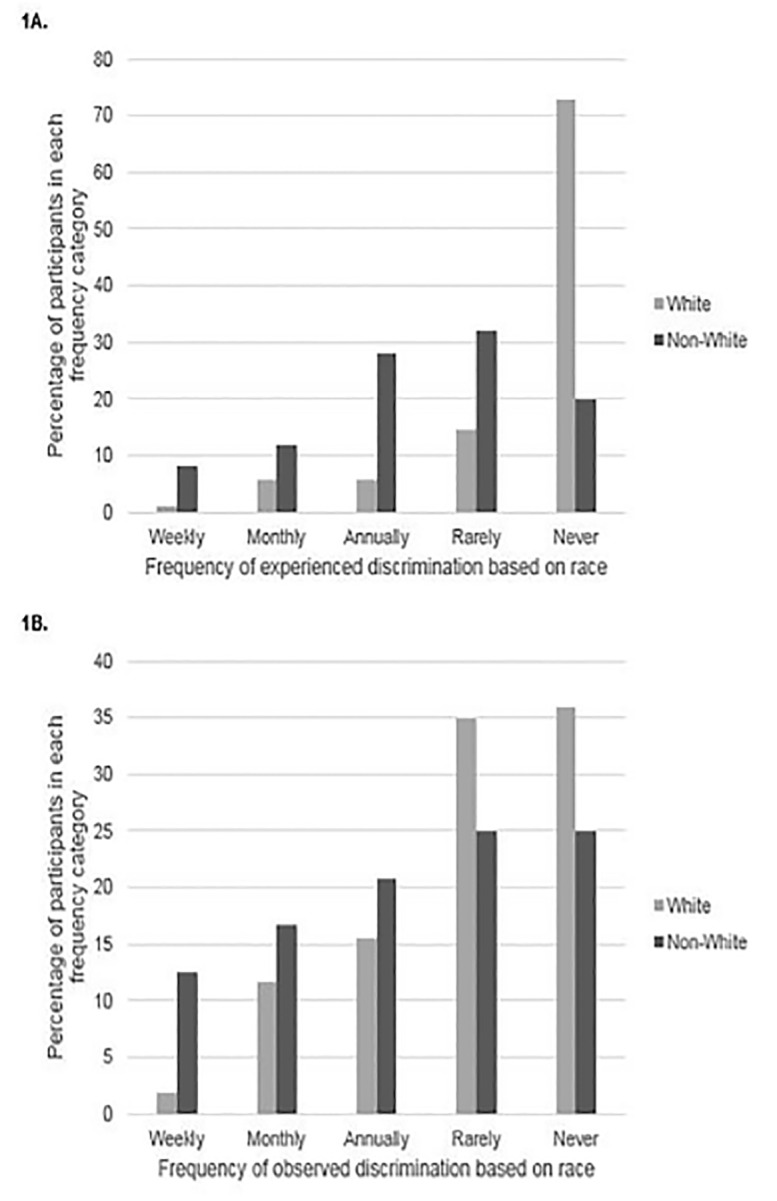
Percentage of participants who (A) experienced or (B) observed race-based discriminatory treatment by racial minority status and frequency.

In our sample, Cronbach’s alpha for the five items of the OGDW scale was 0.84, suggesting an acceptable level of internal consistency. The mean racial OGDW score for all respondents was 9.4 (standard deviation 4.7; 95%, CI, 8.6–10.2), with non-White physicians reporting significantly higher mean racial OGDW scores than their White counterparts (13.4 vs 8.6, respectively; t = −4.502, degrees of freedom [df] = 28.543, p < 0.001, equal variances not assumed; mean difference −5.3, 95% CI for difference, −7.7 – −2.9). Non-White EM faculty were also significantly more likely to report having experienced discriminatory treatment based on race than were White EM faculty (48.0% vs 12.6%, respectively; p < 0.001) (Figure 1). Having experienced discriminatory treatment based on race was significantly associated with higher racial OGDW scores (mean racial OGDW 14.5 vs. 8.5, t = −5.905, df = 31.210, p < 0.001, equal variances not assumed; mean difference −6.0, 95% CI for difference, −8.1 – −3.9). Although non-White physicians were more likely than White physicians to report having experienced race-based discriminatory treatment, the proportion of non-White (50%) and White (29.1%) EM faculty who reported observing race-based discriminatory treatment of another physician was statistically similar (χ^2^ = 3.832, df = 1, p = 0.050) (Figure 1). Having observed race-based discriminatory treatment of another physician was significantly associated with higher racial OGDW scores (12.4 vs 8.2, t = −5.744, df = 131, p < 0.001; mean difference −4.2; 95% CI for difference, −5.6 – −2.7).

Respondent age was not significantly correlated with racial OGDW scores nor observations of discriminatory treatment (r = 0.104, p = 0.454; r = −0.009, p = 0.927, respectively). However, there was an association between age and having experienced race-based discrimination (r = 0.282, p = 0.003), with older respondents reporting more discriminatory experiences. Similarly, respondents’ years in practice were not significantly correlated with racial OGDW scores (r = 0.115, p = 0.189) nor observations of discrimination (r = −0.009, p = 0.922). Yet those respondents with more years in practice reported more race-based discriminatory experiences (r = 0.309, p < 0.001).

**Figure 2:**
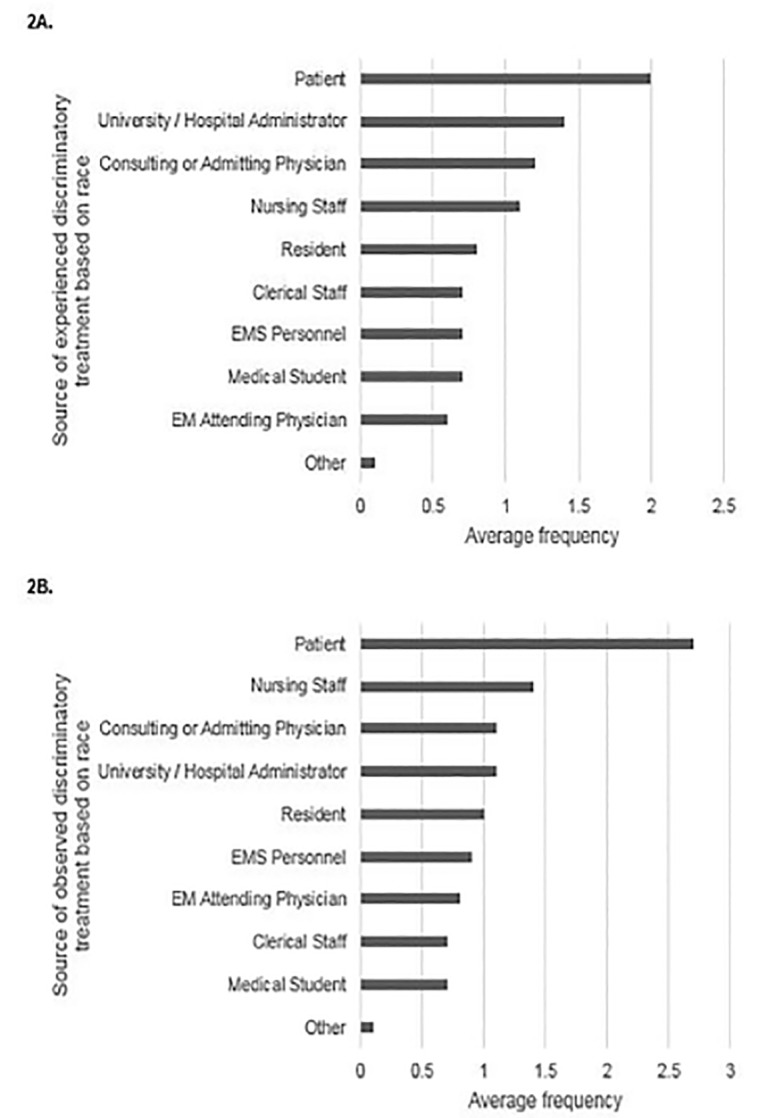
Sources of (A) experienced or (B) observed race-based discriminatory treatment by average frequency.

For those respondents who had experienced discriminatory treatment based on race at least annually, the three most frequent sources of the treatment were patients; university, medical school, or hospital administrators; and consulting or admitting physicians (Figure 2). For those respondents who had observed discriminatory treatment based on race at least annually, the three most frequent sources were patients; nursing staff; and consulting or admitting physicians (Figure 2).

**Figure 3:**
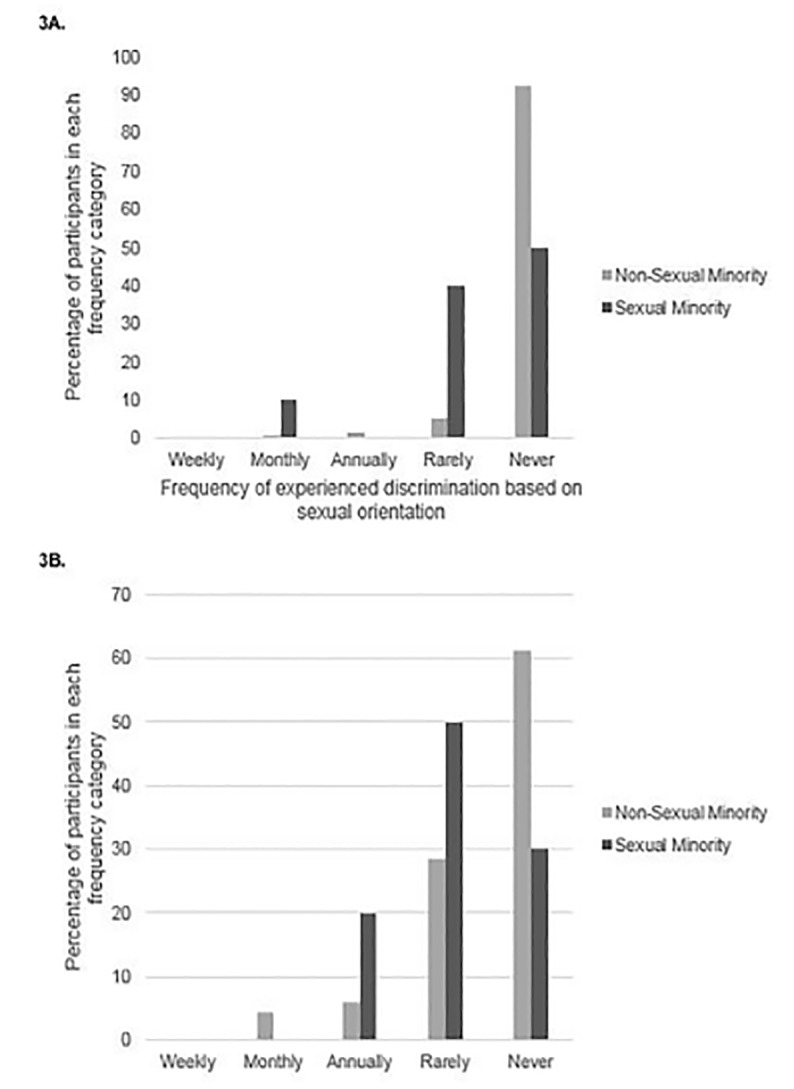
Percentage of participants who (A) experienced or (B)observed sexual orientation-based discriminatory treatment by sexual minority status and frequency.

Cronbach’s alpha for the five items of the OGDW sexual orientation scale was 0.79 in this sample, supporting acceptable internal consistency reliability. The mean sexual minority OGDW score for all participants was 7.1 (SD 3.3, 95% CI, 6.5–7.6), with respondents who identified as sexual minorities reporting significantly higher mean sexual minority OGDW scores than their heterosexual counterparts (11.1 vs 7.1, respectively; t = −2.643, df = 9.461, p = 0.026, equal variances not assumed; mean difference −4.0, 95% CI for difference, −7.3 – −0.6). There were no significant differences between sexual minority and heterosexual respondents in their reports of experiencing discrimination based on sexual orientation, with 10% of minority and 2.5% of heterosexual EM faculty reporting these experiences (p = 0.279) (Figure 3). Having experienced discriminatory treatment based on sexual orientation was significantly associated with higher OGDW scores (mean sexual minority OGDW 12.5 vs 7.34, t = −3.684, df = 128, p < 0.001; mean difference −5.2, 95% CI for difference, −7.9 – −2.4). Sexual minority and heterosexual EM faculty were equally likely to report having observed discriminatory treatment of another physician based on sexual orientation (20% vs 10.3%, χ^2^ = 0.892, df = 1, p = 0.345) (Figure 3). Having observed discriminatory treatment of another physician based on sexual orientation was also associated with higher sexual minority OGDW scores (mean sexual minority OGDW 10.7 vs 7.1, t = −4.917, df = 127, p < 0.001).

There were no consistent relationships between respondent age or years in practice and sexual minority OGDW scores or personal experiences of discriminatory treatment. However, there was an association between both age and years in practice with having observed discrimination of another physician based on sexual orientation (r = 0.227, p = 0.018; r = 0.233, p = 0.008, respectively), with older respondents reporting more discriminatory observations.

**Figure 4:**
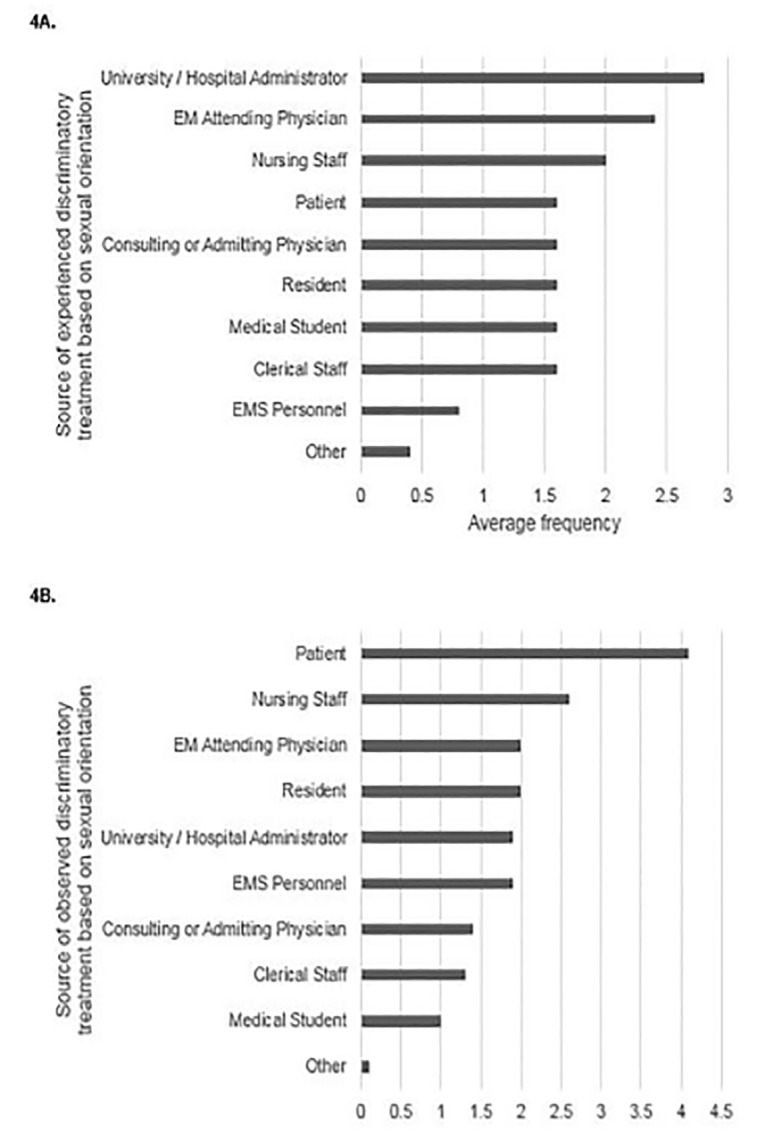
Sources of (A) experienced or (B) observed sexual orientation-based discriminatory treatment by average frequency.

For those respondents who had experienced discriminatory treatment based on sexual orientation at least annually, the three most frequent sources of the discriminatory treatment were university, medical school, or hospital administrators; other EM attending physicians; and nursing staff (Figure 4). For those respondents who had observed discriminatory treatment based on sexual orientation at least annually, the most frequent sources were patients; nursing staff; and other EM attending physicians and residents (Figure 4).

## DISCUSSION

In our study academic emergency physicians who identify as racial or sexual minorities differed significantly when compared to their non-minority colleagues in their perceptions of and experiences with workplace discrimination. Non-White EM faculty were significantly more likely to report experiencing discriminatory treatment based on their race than their White colleagues. This is consistent with studies among physicians across multiple specialties that showed racial minority physicians were significantly more likely to report having experienced racial discrimination both during their medical careers and in their current workplace, including discrimination related to career advancement, punitive behaviors, practice barriers, and hiring barriers.^2^,^27^,^28^ Although we did not ask respondents to detail these reported instances of discrimination, prior research revealed that physicians from racial minorities frequently described encountering microaggressions in the workplace.^29^–^31^

Microaggressions are defined as brief, commonplace, daily, verbal, nonverbal, environmental slights, insults, invalidations, and indignities – intentional or unintentional – directed toward a marginalized group.^32^ There is literature that details the detrimental mental health effects of microaggressions.^31^,^33^ Microaggressions and other forms of workplace discrimination may also have deleterious effects on physicians’ careers. Previous work demonstrated that experiences with racial discrimination, and not physician race, was significantly associated with higher rates of job turnover, with approximately 25% of racial minority physicians reporting that they have left at least one job due to personally experienced workplace discrimination.^6^ Within EM, a national survey of faculty found disparities in rank and leadership positions for physicians of under-represented minority groups.^12^ These data suggest that racial discrimination in the workplace may not only be harmful to the health of minority physicians but it may also significantly impact career trajectories and the retention of a diverse physician workforce.^6^

Although EM faculty who identified as sexual minorities reported more experiences of discriminatory treatment based on their sexual identity compared to their non-sexual minority peers, this was not statistically significant likely due to the limited numbers of respondents who identified as a sexual minority in our sample. Nonetheless, both racial and sexual minority OGDW scores were significantly higher for racial and sexual minority EM faculty than their non-minority counterparts. As expected, having more experiences with and observations of discriminatory treatment based on race and sexual orientation correlated with higher OGDW scores. Interestingly EM faculty regardless of race or sexual orientation were equally likely to report observing discriminatory treatment of another physician based on race or sexual orientation. So although someone may not have direct experience with racial or sexual orientation discrimination, he or she can identify and recognize it when it occurs with another physician.

We did not query respondents about whether they said or did something when they saw these instances of discrimination of another physician, nor did we ask respondents who reported having experienced discrimination whether others intervened on their behalf when there were witnesses. Prior work showed that racial minority physicians were uncomfortable voicing race-related concerns at work,^29^ and among those who did, minority physicians were more likely to find no change in their situation following submission of a complaint compared to their White colleagues.^28^ Similarly, in a national survey of surgery residents, none of the LGBT residents who experienced homophobic remarks reported the event due to fears of reprisal, not wanting to create more “trouble,” or a belief that nothing would be done about the event.^17^ Institutional policies and guidance on how individuals can and should respond to instances of racial or sexual minority discrimination may be helpful. For example, the British Medical Association launched a national campaign in 2001 to inform both patients and providers that racial harassment would not be tolerated in the National Health Service (NHS). This campaign was supplemented by training for all NHS employees focusing on available institutional resources and skills individuals can use to respond to instances of racial discrimination in the workplace.^34^ Other suggested actions to mitigate discriminatory behavior and promote diversity include the identification of best practice efforts to recruit and retain faculty from minority groups, addressing obstacles to advancement, and implementing strategies to promote members of minority groups to positions of leadership.^28^

In our study EM faculty who have been in practice longer were more likely to report having encountered racist behaviors as well as discrimination based on sexual orientation. Prior studies revealed similar findings with regard to racial discrimination^2^ and sexual orientation.^15^ It is unclear whether longer-practicing respondents have had more time in the medical profession to encounter these behaviors, those behaviors were more common in the past, or whether they felt more empowered to report these instances since they may be more established in the field and have less fear of reporting. Future work documenting these trends will be helpful to clarify this question.

Sources of experienced or observed discriminatory treatment based on race were most commonly from patients. This is consistent with recent work that demonstrated that a majority of healthcare providers, including physicians, reported offensive comments from patients about their age, gender, race or ethnicity, weight, or other personal traits.^35^ Physicians from minority backgrounds were more likely to describe discriminatory treatment from patients, with 70% of Black and Asian physicians reporting biased comments from patients.^35^,^36^ Patients were also the most common source of observed discriminatory treatment based on sexual orientation. This may stem from underlying racist or homophobic beliefs that exist within our culture and society. For example, in a 2008 survey of patients, about a third of respondents indicated they would change providers if they found out their provider was gay or lesbian, and a similar number would change practices if they found out gay or lesbian providers were employed there.^37^ Prejudiced comments and behaviors in the healthcare setting are particularly challenging to deal with because physicians have a responsibility to provide appropriate medical care to these patients. Physicians who were subject to discriminatory treatment from patients often experienced an emotional toll that included exhaustion, self-doubt, and cynicism.^38^ Many of these targeted physicians also expressed a need for training on how to deal with biased patients and for clear institutional policies to guide responses.^38^,^39^

The next most common source of experienced or observed discriminatory treatment based on race or sexual orientation was other medical staff. Racism and homophobia within the medical profession have been previously documented.^12^–^14^ Prior work found that racial minority faculty were substantially more likely than majority faculty to perceive racial bias in the workplace, with nearly half reporting experiencing racial discrimination by a work superior or colleague.^2^ Racial minority faculty also described feelings of isolation and invisibility, disrespect with overt and covert bias/discrimination, different performance expectations, devaluing of research on health disparities, the unfair burden of being identified with affirmative action, and responsibility for diversity efforts.^40^ Similarly, among medical students who have experienced anti-LGBT discrimination, the most frequent source originated from fellow medical students.^41^ In a study of surgical residents, the majority of respondents reported having witnessed homophobic remarks by nurses and residents, and about 30% heard similar remarks made by surgical attending physicians.^17^ Among EM residents specifically, 2.5% of trainees reported feeling uncomfortable with other LGBT physicians, and discriminatory LGBT comments were reported from both fellow residents (17%) and faculty (10%).^42^ Unfortunately, discriminatory treatment of sexual minority providers is not uncommon after medical school and residency training. Among practicing physicians who identify as LGBT, approximately 10% reported that they were denied referrals from heterosexual colleagues, 15% had been harassed by a colleague, 22% had been socially ostracized, 65% had heard derogatory comments about LGBT individuals, and 27% had witnessed discriminatory treatment of an LGBT coworker.^13^

Achieving diversity within the physician workforce has been a national priority over the last three decades.^43^,^44^ Most recent data demonstrated that 35.7% of full-time faculty in US medical schools identified as non-White, with 9.7% from under-represented minority (URM) groups.^26^ In EM, approximately 27.0% of full-time faculty identified as non-White, with 10.3% from URM groups. While 7.7% of the 14,254 matriculated US medical students voluntarily identified as lesbian, gay, or bisexual in a 2018 survey, the percentage of practicing physicians who identify as a sexual minority is unknown because neither sexual or gender identity is a required demographic field currently collected by the Accreditation Council for Graduate Medical Education (ACGME) or the AAMC.^45^ Diversity in healthcare is important because it enhances the quality of training for all students and trainees.^46^,^47^ Diversity within medical faculty is particularly significant for the role modeling and mentorship it provides to students and trainees of similar backgrounds.^46^,^47^ A diverse physician workforce has also been shown to reduce healthcare disparities in terms of access and quality.^43^,^48^ In an effort to promote workforce diversity, both the ACGME and the Liaison Committee on Medical Education have detailed elements that residency and medical education programs should have when they are assessed for accreditation.^49^,^50^ Other actions that healthcare organizations can take include bias training, cultural competency and sensitivity training, patient-physician communication training, compensation equity, and workforce diversity initiatives.^48^ As the US population becomes increasingly diverse,^51^ issues regarding physician workforce diversity will remain salient in the future.

## LIMITATIONS

Similar to what we reported previously,^21^ our study population was a convenience sample of EM faculty at six urban academic sites and our results may not be generalizable to practicing emergency physicians in non-urban and non-academic settings. Approximately 40% of eligible subjects responded to the survey and response bias may have played a role in our results. We were unable to compare characteristics of respondents with those of non-respondents due to the anonymous nature of our survey methodology. Therefore, we do not know whether more respondents who identify as racial/ethnic or sexual minorities chose to participate in the study, nor do we know whether their experiences with discrimination or harassment played a role in their study participation. The low numbers of respondents who identify as racial/ethnic and sexual minorities also limited our analyses such that dichotomization of data to White and non-White as well as sexual-minority and non-sexual minority groups were necessary.

The OGDW scale was originally intended to measure perceptions of gender discrimination, and its validity in measuring racial and sexual minority discrimination has not been examined. Although our questions measuring experiences and observations of racial and sexual orientation discrimination were modeled after prior work and have face validity, their reliability as well as criterion and construct validity have not been established. In addition, our results were based on physicians’ self-reports of perceived or experienced discrimination, and thus we were unable to corroborate respondents’ self-reported experiences and observations with racial or sexual orientation discrimination. Nonetheless, researchers have found that self-reports of discrimination are accurate and reliable when validated against other data sources.^52^ Finally, our study did not use qualitative methods to explore in-depth our respondents’ varied and multi-faceted experiences with workplace discrimination that may provide additional context to our survey findings.

## CONCLUSION

Racial and sexual minority EM faculty perceived more discrimination based on race and sexual orientation, respectively, in their workplace than their non-minority counterparts. Perceptions of discrimination were associated with direct experience with and observations of discriminatory treatment. Although non-White EM faculty were more likely to experience racial discrimination than their White colleagues, both groups were similar in their observations of discriminatory treatment of another physician based on race. Similarly, EM faculty regardless of sexual orientation were similar in their observations of discriminatory treatment of another physician based on sexual orientation. Future work examining the prevalence and characteristics of racial and sexual orientation discrimination in a larger and more diverse sample of emergency physicians is necessary.

## Supplementary Information



## Figures and Tables

**Table 1:** Participant characteristics in emergency medicine faculty racial and sexual orientation discrimination survey.

Characteristics	Participants (N = 141)	
Age (years)		
≤39	52	(47.3)
40–49	41	(37.3)
50–59	16	(14.5)
≥60	1	(0.9)
Years out of training		
1–5	33	(25.2)
6–10	40	(30.5)
11–15	26	(19.8)
16–20	15	(11.5)
≥21	17	(13.0)
Gender		
Male	80	(61.1)
Female	51	(38.9)
Race/Ethnicity		
White	104	(79.4)
Black/African American	6	(4.6)
Hispanic/Latino	5	(3.8)
Asian/Pacific Islander	12	(9.2)
American Indian/Alaska Native	2	(1.5)
Other	2	(1.5)
Sexual Orientation		
Straight / Heterosexual	120	(90.9)
Gay / Lesbian / Homosexual	8	(6.1)
Bisexual	2	(1.5)
Decline to answer	2	(1.5)

Data are reported as n (%).
