# Supplementary material for: Academic Emergency Medicine Faculty Experiences with Racial and Sexual Orientation Discrimination
Source: West J Emerg Med. 2020 Aug 21;21(5):1160–9. doi: 10.5811/westjem.2020.6.47123 (PMC7514380; doi:10.5811/westjem.2020.6.47123)
Supplement: Supplementary file 1 [file wjem-21-1160-s001.docx]

**Appendix: Survey Questions Related to Racial and Sexual Orientation Discrimination**

Racial/Ethnic Discrimination Questions

How strongly do you agree with the following statements about your current place of work? (scale of 1-5, with 1 = strongly disagree, 3 = neutral, 5 = strongly agree)

1. I have been treated unfairly at work because of my race or ethnicity.
2. The people I work with sometimes make racist statements and/or decisions.
3. I feel that some of the policies and practices of this organization are racist.
4. At work, I sometimes feel that my race or ethnicity is a limitation.
5. At work, I do not get enough recognition because of my race or ethnicity.

----------

1. In my current institution, I’ve experienced discriminatory treatment based on my race or ethnicity:

- Weekly
- Monthly
- Annually
- Rarely
- Never

If Weekly, Monthly, Annually:

6.A. How often have you experienced discriminatory treatment based on your race or ethnicity from the following sources?: (for each source, choice of weekly, monthly, annually, rarely, never)

- University / Medical School or Hospital administrator
- Consulting or admitting physician
- EM attending physician
- Resident
- Medical student
- Nursing staff
- Clerical staff
- EMS personnel
- Patient
- Other: __________

6.B. To what extent have these experiences had a negative effect on your confidence in yourself as a professional? (1-5 Likert scale, 1 = not at all, 5 = greatly)

6.C. To what extent have these experiences negatively affected your career advancement? (1-5 Likert scale, 1 = not at all, 5 = greatly)

1. In my current institution, I’ve observed discriminatory treatment of another physician based on race or ethnicity:

- Weekly
- Monthly
- Annually
- Rarely
- Never

If Weekly, Monthly, Annually:

7.A. How often have you observed discriminatory treatment of another physician based on race from the following sources?: (weekly, monthly, annually, rarely, never)

- University / Medical School or Hospital administrator
- Consulting or admitting physician
- EM attending physician
- Resident
- Medical student
- Nursing staff
- Clerical staff
- EMS personnel
- Patient
- Other: __________

**Sexual Orientation Discrimination Questions:**

How strongly do you agree with the following statements about your current place of work? (scale of 1-5, with 1 = strongly disagree, 3 = neutral, 5 = strongly agree)

1. I have been treated unfairly at work because of my sexual orientation.
2. The people I work with sometimes make anti-LGBTQ statements and/or decisions.
3. I feel that some of the policies and practices of this organization are anti-LGBTQ.
4. At work, I sometimes feel that my sexual orientation is a limitation.
5. At work, I do not get enough recognition because of my sexual orientation.

----------

1. In my residency training, I’ve experienced discriminatory treatment based on my sexual orientation:

- Weekly
- Monthly
- Annually
- Rarely
- Never

If Weekly, Monthly, Annually:

13.A. How often have you experienced discriminatory treatment based on your sexual orientation from the following sources?: (for each source, choice of weekly, monthly, annually, rarely, never)

- University / Medical School or Hospital administrator
- Consulting or admitting physician
- EM attending physician
- Resident
- Medical student
- Nursing staff
- Clerical staff
- EMS personnel
- Patient
- Other: __________

13.B. To what extent have these experiences had a negative effect on your confidence in yourself as a professional? (1-5 Likert scale, 1 = not at all, 5 = greatly)

13.C. To what extent have these experiences negatively affected your career advancement? (1-5 Likert scale, 1 = not at all, 5 = greatly)

1. In my residency training, I’ve observed discriminatory treatment of another physician based on sexual orientation:

- Weekly
- Monthly
- Annually
- Rarely
- Never

If Weekly, Monthly, Annually:

14.A. How often have you observed discriminatory treatment of another physician based on sexual orientation from the following sources?: (weekly, monthly, annually, rarely, never)

- University / Medical School or Hospital administrator
- Consulting or admitting physician
- EM attending physician
- Resident
- Medical student
- Nursing staff
- Clerical staff
- EMS personnel
- Patient
- Other: __________

Demographics

1. What is your age?
2. How many years have you been practicing since completion of residency?

- 1-5 (=1)
- 6-10 (=2)
- 11-15 (=3)
- 16-20 (=4)
- 21+ (=5)

1. What is your race or ethnicity? (check all that apply)
   - - American Indian / Alaska Native (=1)
     - Asian (=2)
     - Black / African American (=3)
     - Hispanic / Latino (=4)
     - Native Hawaiian / other Pacific Islander (=5)
     - White (=6)
     - Other (please specify) (=7)
2. What is your gender identity?

- Male (=1)
- Female (=0)
- Transgender
- Another gender identity

1. What is your sexual orientation? (check all that apply)

- Straight / Heterosexual (=0)
- Gay / Lesbian / Homosexual (=1)
- Bisexual (=2)
- Queer (=3)
- Questioning (=4)
- Another sexual orientation (=5)
- Decline to answer (=6)
